# Ecotoxicological Assessment of “Glitter” Leachates in Aquatic Ecosystems: An Integrated Approach

**DOI:** 10.3390/toxics10110677

**Published:** 2022-11-09

**Authors:** Manuela Piccardo, Francesca Provenza, Serena Anselmi, Monia Renzi

**Affiliations:** 1 Dipartimento di Scienze della Vita, Università di Trieste, 34127 Trieste, Italy; 2 Bioscience Research Center, Via Aurelia Vecchia 32, 58015 Orbetello, Italy; 3 CoNISMa, Consorzio Interuniversitario per le Scienze del Mare, Piazzale Flaminio 4, 00196 Roma, Italy

**Keywords:** battery of bioassays, freshwater, saltwater, microplastics, chemical risk, integrated approach, *Allivibrio fischeri*, algae, *Daphnia magna*, *Paracentrotus lividus*

## Abstract

The most worrisome fraction within plastic pollution is that of microplastics (MP). A category of MP almost completely ignored is that of glitter. The objective of this study is to test the toxicity of nine types of glitter leachate (3 soak times: 3, 90 and 180 days) on model organisms in freshwater (*Allivibrio fischeri*, *Raphidocelis subcapitata*, *Daphnia magna*) and saltwater (*Allivibrio fischeri*, *Phaeodactylum tricornutum*, *Paracentrotus lividus*). An integrated approach was applied to obtain the percentage of ecotoxicological risk. The results show that (i) photosynthesizing primary producers are the most sensitive trophic level; (ii) algae transitioned from growth inhibition to biostimulation; (iii) *D. magna* showed higher sensitivity after 48 h compared to 24 h; (iv) *A. fischeri* responded more strongly in saltwater than in freshwater. The integrated data show a greater risk associated with the marine environment, with the highest risk for glitters that are hexagonal and composed of poly-methyl-methacrylate. Our multivariate analysis shows that the toxicity of plastic leaching is a complex phenomenon that depends on the sensitivity of the species, in some cases on the soaking time and on the medium, and is not clearly linked to the polymer type, the contact area or the colors of the particles.

## 1. Introduction

In 2020, 367 million tons of plastic were produced worldwide, 15% of which came from Europe [1]. Most of the production (40.5%) was in plastic packaging, construction (20.4%) and motor vehicles (8.8%). In Europe, 34.6% of waste produced is recycled, 42% is destined for energy production, and the remaining 23.4% is disposed of in landfills [1]. The ever-increasing global production of plastics over the last seven decades has led to their widespread distribution in the environment, and they have become an important geological indicator of the Anthropocene [2,3]. Due to poor waste management and without improvement of waste management infrastructure, the cumulative amount of plastic waste entering the oceans is expected to reach 250 million tons by 2025, an order of magnitude increase compared to 2015 [4]. Plastic debris has been detected at all latitudes from the poles [5,6] to the equator [7], in surface waters [8,9,10] and in the deep oceans [11,12,13], and represents a form of pollution that may pose a threat to both freshwater [14,15,16] and marine environments [17].

The most worrisome fraction within plastic waste is that of microplastics (MP), plastic particles ranging in size from 20 to 5000 µm (as defined by the MSFD Technical Subgroup on Marine Litter, [18]). The danger of MP lies in their bioavailability to a wide range of organisms and thus their toxicity through physical clogging, as well as their ability to act as a Trojan horse by transferring and mobilizing the co-pollutant cocktail associated with plastic production or other co-pollutants, pathogens or alien species present in the surrounding medium [3,19,20,21]. Various substances are used in the production of plastics: plasticizers (bisphenol A, BPA; phthalates such as diisobutyl phthalate, DIBP), catalysts (metals), flame retardants (hexabromocyclododecane, HBCD), pigments, dyes, etc. [3]. Some of these substances have been shown to have harmful effects and are classified as carcinogenic or endocrine disruptors (e.g., phthalates [22]). Because these substances are only weakly associated with the surface of the microplastic, they can be released into the environment [23] and, if ingested, can spread into organisms and have effects at different levels of biological organization [24].

MP can either result from the fragmentation of large objects (secondary microplastics) or they can enter the environment directly as pellets, beads, and fibers (secondary microplastics) [19]. Some of these categories, such as microspheres in personal care products, have been withdrawn from the market in many European countries, including Italy (2018 Budget Law, https://www.gazzettaufficiale.it/eli/id/2018/12/31/18G00172/sg; accessed on 10 October 2022). However, there is another category of primary MP that is almost completely ignored today: glitter. The term glitter refers to an assortment of small, flat, reflective particles made of a plastic polymer coated with a metal such as aluminum, titanium, iron, or bismuth, which gives it a high reflectivity [25]. Commercially available glitter ranges in size from 50 to 6350 μm, with the most common size being about 200 μm [26]. Glitter is produced by the ton each year worldwide and is mainly used in makeup and craft materials, but also in a variety of activities such as face wash, furniture, toys, clothing, and accessories [27]. Plastic glitter comes in all colors and in various shapes (in precision-cut pieces of uniform size). Glitter can enter water bodies through sewage and landfill runoff [27]. Glitter particles have been found in surface waters and sediment samples from an Indian estuary [28], in sediments from Lake Ontario [29], and in urban dust from several Iranian cities [30,31,32,33]. Even though many glitter particles are removed by wastewater treatment plants [34], a large amount of these particles still enters the ocean. Raju et al. (2020) [35] estimated the daily input of glitter particles from a wastewater treatment plant to be 2.7–3.0 × 10^7^ particles/day.

Inasmuch as it is waste in the environment, glitter accumulates as a pollutant in the environment and interacts with biota. Studies on the ecotoxicity of glitter particles are scarce. One is the study by Green et al. (2021) [36], which investigated the effects of glitter from conventional (PET) or alternative materials (modified regenerated cellulose, mica, or synthetic mica) on biodiversity and ecosystem functioning of freshwater, lotic habitats. Overall, the results suggest that both conventional and alternative glitters can have ecological impacts on aquatic ecosystems by increasing the abundance of New Zealand mud snails (*Potamopyrgus antipodarum*) twofold and decreasing duckweed (*Lemna minor*) root length and phytoplankton biomass. However, despite the multimaterial nature of glitter particles, nothing is known about the chemical risk associated with glitter leachate to aquatic ecosystems.

The aim of this study is to test the toxicity of nine types of glitter purchased in the handicraft market, differentiated by different shapes and sizes (and therefore different contact areas with the aqueous medium), colors (different dyes) and chemical compositions (poly-methyl-methacrylate PMMA, polyethylene PE, polyamide PA). Thus, for the first time, chemical risk was studied for model organisms in freshwater (bacteria *Aliivibrio fischeri*, algae *Raphidocelis subcapitata*, crustaceans *Daphnia magna*) and saltwater (*Aliivibrio fischeri*, algae *Phaeodactylum tricornutum*, sea urchin *Paracentrotus lividus*) to provide the first useful basis for ecotoxicological risk assessment. In particular, glitter was used to prepare aqueous extracts obtained with three different soaking times (3, 90 and 180 days) to better investigate temporal dynamics. Finally, our results were used to calculate an integrated toxicity test battery index (hereafter TBI) and the corresponding ecotoxicological risk percentage (%*R*) as listed in the ISPRA manual (2011) [37] and previously calculated by Schiavo et al. (2019) [38] for the ecotoxicological assessment of leachates from plastic pellets in freshwater matrices.

## 2. Materials and Methods

### 2.1. Glitter Characterization

The glitters were purchased in physical stores, are in the form of powder, and were originally intended for the decorative arts industry (e.g., purpurine), the home furnishings industry (e.g., as an additive for paints), and the cosmetics industry (glitter for hair gel). The leachates for the ecotoxicological test were prepared from nine types of glitter microparticles that differed in shape (hexagons, stars, and rectangles), size (from 221.64 to 3047.83 μm mean maximum length), and color (silver, green, orange, purple, yellow, and pink) (see Table 1). The colors of the glitter were determined before and after the leaching test using the CLM-194 portable colorimeter (EOPTIS). The color values determined by the colorimeter are displayed in native CIELAB coordinates (L * a * b). The 1976 CIELAB color space is a color space defined in 1976 by the International Commission on Illumination (abbreviated CIE) and was intended to be a perceptually uniform color space in which a given numerical change corresponds to a similar perceived color change. In this color space, the distance between two points indicates approximately how different the colors are in terms of luminance (L), chroma (a), and hue (b). In other words, luminance is brightness, chroma is saturation, and hue is tint (in terms of blue and yellow). Finally, the differences in color values were used as evidence of color output.

### 2.2. Leachate Preparation

The leachates were prepared by immersing 100 mg of each glitter in 1 L of artificial seawater (ASW) and ISO FRESHWATER (ISO FW) (see Supplemental Materials for the composition of these solutions). This concentration was chosen because it is the reference concentration in numerous regulations such as REACH EU Regulation No. 1907/2006 and the OECD Guidelines for the Testing of Chemicals (Section 3, Ready Biodegradability Test [39]) and has been used in numerous studies with plastic particles [40,41,42,43].

Since a large number of containers were needed (i.e., 66), the dispersions were prepared in plastic bottles. To account for a possible contribution of plastic, two different negative controls (ASW only or ISO FW) were considered: the control in glass bottles (hereafter referred to as CN/GL) and the control in plastic bottles (hereafter referred to as CN/PL). The experiment included three different soaking periods: 3, 90 and 180 days (hereafter referred to as TIME 1, 2 and 3, respectively). During this time, samples were shaken daily at 40 rpm for 1 h and stored at room temperature (22–25 °C) in a dark place.

At the end of the soaking periods, the dispersions were filtered with a vacuum pump at 0.45 microns (mixed cellulose ester filter membrane), and the liquid was used for the ecotoxicological tests. The glitters on the filters were otherwise observed under a stereomicroscope to detect any morphological changes and analyzed again with the colorimeter.

### 2.3. Toxicity Tests

Two different standardized test batteries were used to test the toxicity of glitter leachate to freshwater and marine environments. For freshwater, the battery consisted of *Aliivibrio fischeri* (acute toxicity test; UNI EN ISO 11348-3:2019; endpoint: inhibition of bioluminescence), *Raphidocelis subcapitata* (chronic toxicity test; UNI EN ISO 8692:2012; endpoint: growth inhibition), and *Daphnia magna* (acute toxicity test; UNI EN ISO 6341:2013; endpoint: immobility). For the marine environment, the battery consisted of *Aliivibrio fischeri* (acute toxicity test; UNI EN ISO 11348-3:2019; endpoint: inhibition of bioluminescence), *Phaeodactylum tricornutum* (chronic toxicity test; UNI EN ISO10253:2017; endpoint: growth inhibition) and *Paracentrotus lividus* (acute toxicity test; Chapman et al. 1995 + ISPRA Quaderni Ricerca Marina 11/2017; endpoint: abnormal larvae). For more information on the protocols used, see Supplementary Materials (Supplementary Material, Table S1: Toxicity tests performed on glitter leachates).

#### 2.3.1. Test on Bacteria

Biological responses to bacteria were tested on the species *Aliivibrio fischeri* using the Microtox^®^ (Ecotox) photometer and lyophilized bacteria purchased from Microbiotests Inc. (Kleimoer 15, 9030 Gent, Belgium, Germany). The percentage of inhibition of natural bioluminescence was measured after 15 and 30 min of exposure to 90% of the concentration of the leachate in fresh and salt water, using two experimental replicates.

#### 2.3.2. Test on Algae

Biological responses to marine algae were tested on the microalga *Phaeodactylum tricornutum*. Biological responses to freshwater algae were tested on the microalga *Raphidocelis subcapitata*. Percent growth inhibition (I%) after 72 h of exposure was performed on leachates. In both cases, cell density was measured spectrophotometrically and calculated by light absorption at a wavelength of 670 nm. The response of the spectrophotometer was calibrated using a curve of cell density versus absorbance generated with the algal material tested by counts in the Burker chamber at each of the 10 points of scalar dilution of 10^6^ cells/mL of material. The tests were performed in triplicate at 20 ± 2 °C.

#### 2.3.3. Test on Crustaceans

Biological responses to crustaceans were tested on *Daphnia magna*. Resting-eggs of Daphnia magna were rinsed with ISO FW and transferred to a Petri dish with additional ISO FW. They were left to hatch in an incubator at 21 ± 1 °C (18:6 h light: dark) for 3 days. Exposure studies were performed in the dark at 20 ± 2 °C. Exposure solutions (10 mL) were placed in 10 mL multi-wells, and 5 neonates (<24 h post hatching) were placed in each well and kept in the dark. The neonates were not fed for the duration of the experiment. After 24 and 48 h, the immobility (mortality) of the animals in the container was recorded. Animals that were unable to swim within 15 s of gently shaking the test vessel were considered immobile. All exposure experiments were performed in quadruplicate.

#### 2.3.4. Test on Echinoderms

Biological responses to echinoderms were tested in the sea urchin *Paracentrotus lividus*. Mature sea urchin specimens were captured in a natural marine area (Tuscany) and kept in captivity until the start of the experiment. Exposure tests were performed under fasting conditions as indicated in the method. The percentage of abnormal larvae was calculated on 100 randomly selected *plutei* in each experimental series (*n* = 3). Larvae were considered abnormal if they exhibited arrested development, all arms were missing or of different lengths, there were extra arms with crossed lateral bars, asymmetric body width was detected, or other anomalies appeared as listed in the literature [44].

### 2.4. Quality Assurance and Quality Control

Toxicity tests were performed by the authors in a certified laboratory (UNI EN ISO 9001:2015; UNI EN ISO 17025:2005) to ensure the quality of the data generated. QA/QC assays were performed as described in the previously mentioned reference methods. Positive controls were performed by direct exposure of the tested species to standard toxicants: *A. fischeri* was exposed to 3,5′-dichlorophenol; *P. tricornutum*, *R. subcapitata* and *D. magna* were exposed to K_2_Cr_2_O_7_; *P. lividus* was exposed to Cu(NO_3_)_2_ * 3H_2_O; all were within the acceptance criteria defined by the standard methods.

### 2.5. Statistical Analyses

All data are expressed as mean effect percentage ± standard deviation. In cases where the negative control in plastic (CN/PL) exceeded the threshold established by the method, results were normalized relative to controls using the Abbott formula (X − Y)/(100 − Y) × 100, where X = % of effect in treatment sample; Y = % of effect in control sample (CN/PL).

The reference thresholds of effect (indicating the minimum change considered biologically significant for each experimental condition) were taken from the protocols used (see Section 2.3, “Toxicity tests”) and the literature [37,45] and corresponded to: 15% for *A. fischeri*; 20% for *D. magna;* 10% for *R. subcapitata* and *P. tricornutum*; and 20% for *P. lividus*.

Two-way ANOVA (significance of observed differences between the “time” and “polymer” factors within treatments) was performed for the ecotoxicity dataset. Differences were considered significant when the *p*-level was <0.05. Data were analyzed using GraphPad Prism (GraphPad Software, version 6.01, San Diego, CA, USA, www.graphpad.com, accessed October 2022).

Multivariate analyses were performed using Primer v 7.0 (Primer-E Ltd., Plymouth Marine Laboratory, Plymouth, UK) according to the methods described in Clarke and Warwick (1999) [46]. The Euclidean distance matrix was calculated for the effect data normalized as percentages. The factors tested for mean effects were: Time (three levels, fixed: 1, 2 and 3) and Polymer (three levels, fixed: PMMA, PA and PE). A (normalized) environmental data set composed of the variables “contact area”, “∆*E*”, “∆*L*”, “∆*a*” and ”∆*b*” was used to determine their relative importance for the biological variables (DistLM function, i.e., distance-based linear models).

∆*E* is a unit of measurement used to calculate and quantify the difference between two colors (one a reference color, the other a sample color trying to match it) based on L * a * b * coordinates. ∆*E* was calculated according to the following formula:

$$∆E76=∆E=\sqrt{{\left(L_{a}-L_{b}\right)}^{2}+{\left(a_{a}-a_{b}\right)}^{2}+{\left(b_{a}-b_{b}\right)}^{2}}$$

where *L* is the luminance, *a* is the chromaticity, and b is the hue. Subscript a and b represent after and before the leaching test, respectively. ∆*E* is measured on a scale from 0 to 100, where 0 represents a small color difference and 100 represents complete distortion.

### 2.6. Data Integration

The data integration used in this study started from the quantitative assessment proposed by Hartwell and successively modified by ISPRA et al. (2011) [37]. It calculates the “ecotoxicological risk”, weighting the type of endpoint observed, the type of environmental matrix analyzed and the level of agreement of the test results. To calculate the TBI, the percentage of effect (%*E*) on each endpoint was corrected to obtain the score test endpoint (*SEi*) according to the following formula:

SEi=%E (M∗S) SCF

where *SCF* (Statistical Correction Factor) = Student *t*-test differences between samples and control. Values 0, 1, 2, 3 and 4 were attributed to *SCF*, corresponding to no effect (*p* > 0.05), biostimulation (*p* < 0.05), high biostimulation (*p* < 0.01), toxicity (*p* < 0.05) and high toxicity (*p* < 0.01); matrix (*M*) was set as 2 for the leachate samples; severity (*S*) was set as 2 for bioluminescence, 3 for algal growth, 4 for development and 5 for mortality.

*SEi* is expressed as percentage (%*SEi*) on a scale of 0–100 relative to the test battery used, as follows:

%SEi=SEi [(%Em)/SEmax)]

where %*Em* = maximum percentage of observed effect corresponding to the maximum achieved MS, and *SEmax* is the maximum calculated score test endpoint.

The toxicity test battery integrated index (*TBI*) is calculated using the following formula:

%TBI=(∑%SEi)/N

where N = number of endpoints (= 3).

The TBI can be used to calculate the percent ecotoxicological risk (%*R*), as follows:

%R=[%TBI∗(∑SEi+C)]/%Sei

where $C={\left(\frac{N}{2-X}\right)}^{3}$, with X = number of statistically non-significant endpoints.

Ecotoxicological risk is defined as follows: non-significant (*R* ≤ 5%), moderate (5 < *R* ≤ 20%), high (20 < *R* ≤ 50%), very high (*R* > 50%).

## 3. Results

### 3.1. Ecotoxicological Responses

#### 3.1.1. Impact on Freshwater System

**Figure 1:**
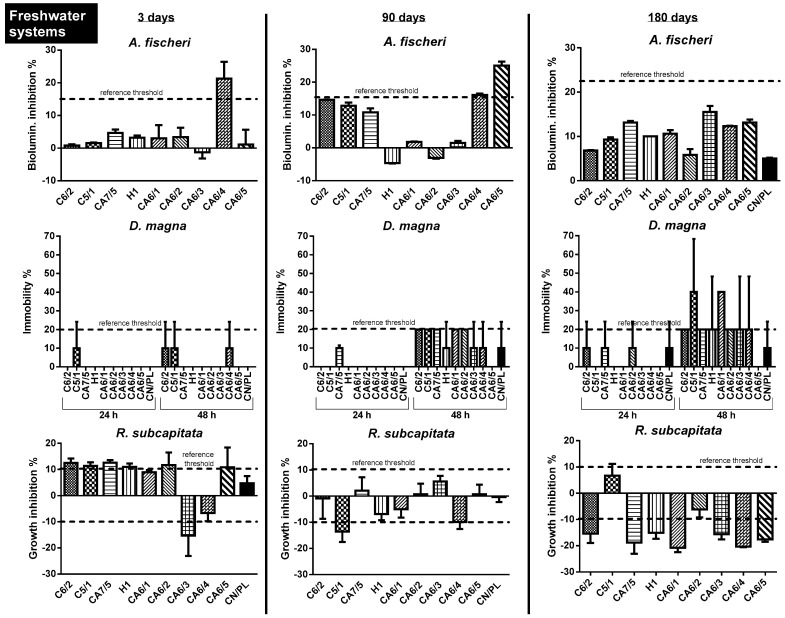
Biological responses of the different model species exposed to glitter leachate in freshwater according to the different soaking times: 3, 90 and 180 days. The dashed line refers to the reference threshold established by the method (15% for *A. fischeri*; 20% for *D. magna*; 10% for *R. subcapitata)*. Negative controls in plastic containers (CN/PL) are shown in black-fill pattern (if not present, data were normalized using the Abbott formula; see Section 2.5).

The effects on the freshwater system were measured using a multispecies battery of *A. fischeri*, *D. magna* and *R. subcapitata* and are shown graphically in Figure 1.

Leachate with a soaking time of 3 days resulted in inhibition of bacterial bioluminescence above the threshold in one case (i.e., CA6/4, 21.3%), inhibition of algal growth in most cases except CA6/4 (−6.67%), but also biostimulation of *R. subcapitata* in the case of CA6/3 (−15.26%). Inhibition of bacterial bioluminescence rarely exceeded the threshold after 90 and 180 days of soaking, as did the percentage of mortality in *D. magna*. If the effect on the algae was still within the thresholds at time 2, it worsened after 180 days, but moved in the opposite direction, i.e., biostimulation, with values ranging from −15.1% to 20.8%.

Data analysis using the TBI approach shows that in most cases there is no ecotoxicological risk, with the exception of CA6/4 and CA6/5 at time 1 and 2 (%*R* = 6% and 8.5%, respectively) (Table 2).

#### 3.1.2. Impact on Marine System

**Figure 2:**
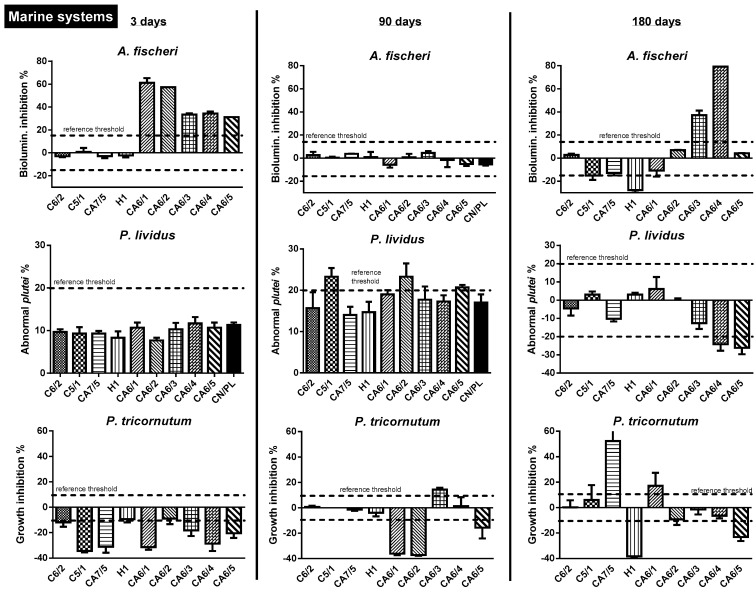
Biological responses of the different model species exposed to glitter leachate in saltwater according to the different soaking times: 3, 90 and 180 days. The dashed line refers to the reference threshold established by the method (15% for *A. fischeri*; 20% for *P. lividus*; 10% for *P. tricornutum)*. Negative controls in plastic containers (CN/PL) are shown in black-fill pattern (if not present, data were normalized using the Abbott formula; see Section 2.5).

The impact on the marine system was measured using a multispecies battery of *A. fischeri*, *P. lividus* and *P. tricornutum* and is shown graphically in Figure 2.

A considerable percentage of bioluminescence inhibition in *A. fischeri* was measured at time 1 for the group of glitters from CA6/1 to CA6/5 (values ranging from 31.3% to 61.2%). At time point 2, the response returned to the accepted values and then returned at time point 3 with a dual response: inhibition for CA6/3 and CA6/4 (37.2% and 79.3%, respectively) and biostimulation in C5/1 and H1 (−15.1% and −27.2%, respectively). In *P. lividus*, thresholds were observed for C5/1, CA6/2 and CA6/5 at time 2 and for CA6/4 and CA6/5 at time 3. For *P. tricornutum,* thresholds were exceeded several times, with inhibition in some cases (up to 52.2%) but biostimulation in the majority of cases (maximum effect in H1, time 3, −38.2%).

Data analysis using the TBI approach showed a moderate ecotoxicological risk for all types of glitter except C6/2 and H1 and a high risk (%*R* = 21.3) for CA6/2 (Table 3).

### 3.2. Species Sensitivity

The species used for the toxicity tests reacted differently to the samples tested and showed varying sensitivity. Table 4 shows the number of times stress thresholds were exceeded, specifically by species, with no internal differentiation between time points. The score can vary from a minimum of 0 to a maximum of 3 (corresponding to the three time points). The most sensitive trophic level was that of photosynthetic primary producers, which responded to treatment in two ways: in some cases by reducing growth rate, in others by increasing it. The second most sensitive species was *A. fischeri* followed by the larval stages of the small planktonic crustacean *D. magna* and the *plutei* of the sea urchin *P. lividus*.

### 3.3. Color Differences after the Soaking Time

The total differences (∆*E*) observed in time 1 range from 2.6 to 42.4, peaking for CA6/2 (Table 5). For the most dangerous type of glitter, a variation of −41.7 in chroma (∆*b*) was the most influential overall difference (*E*) parameter. Negative values of *∆b* indicate a bluer effect. The total differences observed in time 3 range from 2.9 to 30.9, peaking at CA6/2. Regarding the other hazardous glitter types, the brightness variation at CA7/5 was the parameter with the greatest influence on the total difference (∆*E*), which can be attributed to the leaching of the surface reflective layer, as shown by the microscopic observations (Supplementary Materials, Figure S1: CA7/5 type glitter: one of these glitters has the typical outer reflective layer, another is completely colorless). With respect to CA6/3 and CA6/4, hue variation was the most influential parameter overall. Further details on the measured color variations and how these variations are correlated with ecotoxicological responses are discussed in more detail in the multivariable analysis (Section 3.4).

### 3.4. Multivariate Analysis

A two-way analysis ANOVA (Supplemental Materials, Table S2: *p* values of 2-way ANOVA) was performed for marine and freshwater datasets to determine a possible contribution of the factors “time” and “polymer” to the ecotoxicological responses. Multivariate analysis confirmed the significance of the factor of time in determining the differences in the species *R. subcapitata* (*p*-value = 0.0018) and *D. magna* (*p*-value = 0.0296). In marine systems, the algal assay showed an effect of growth biostimulation independent of the time factor in many cases (*p*-value = 0.4383). However, the “polymer” factor was never statistically significant.

**Figure 3:**
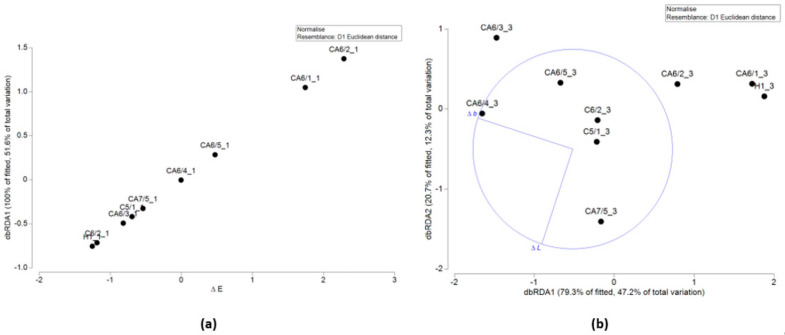
Multivariate analysis performed with Primer7 to determine possible correlation between particle contact area and color variations on ecotoxicological responses in salt water: (**a**) time 1 (i.e., 3 days); (**b**) time 3 (i.e., 180 days).

The integrated TBI approach showed that the ocean was the most vulnerable context at time 1 and 3. Therefore, for these two scenarios, the multivariate analyses were deepened to determine the possible importance of some variables, such as particle contact area and color variations, on the ecotoxicological responses. At time 1, the marginal test of the DistLM model showed that the ∆*E* value (*p* value = 0.002, *R*^2^ = 0.94) was statistically significant and could explain 51.6% of the total variability (Figure 3a). As can be seen from the graph, a higher ∆*E* value is associated with a higher percentage risk: CA6/1 (%*R* = 10.0) and CA6/2 (%*R* = 21.3). At time 3, the most influential variable was the “hue” component (∆*b*), corresponding to a *p*-value = 0.006 (*R*^2^ = 0.79). The combination of ∆*b* and ∆*L* shown in Figure 3b partially explains the greater risk associated with CA7/5 (%*R* = 15.8), CA6/3 (%*R* = 7.3), and CA6/4 (%*R* = 18.4) glitters. A greater toxicity of CA6 appears to be associated with variations in hue, with values tending toward yellow, suggesting possible release of dyes into the aqueous medium. The toxicity of CA7/5, on the other hand, is associated with large variations in brightness (∆*L*), most likely due to the release of the reflective surface layer, as already explained in Section 3.3.

## 4. Discussion

A double-negative control (only water in plastic and glass bottles) was performed to exclude a possible contribution due to the composition of the container. The differences in the ecotoxicological responses of the negative control stored in glass and plastic bottles are shown in Figure S2 (Supplementary Materials, Figure S2: Comparison between the ecotoxicological responses recorded in the two different negative controls). In most cases, the responses are similar. Larger differences (>100%) are reported from *A. fischeri* in saltwater at time 1 and from *P. lividus* at time 3 (>25%). Nevertheless, data correction was performed with the Abbott formula using the values of the plastic control (CN/PL), so any measure differences can be related to the presence of glitters.

The glitter in freshwater behaved differently regardless of the polymer. However, a small temporal gradient was observed especially in the algal test and in *D. magna*. *R. subcapitata* passed from inhibiting algal growth to biostimulation. Therefore, it is suspected that the composition of elutriates changed between time 1 (3 days) and time 3 (180 days). Consequently, *R. subcapitata* may respond in different and even opposite ways by inhibition or biostimulation (hormesis). Thus, after 180 days, trace elements (e.g., metals) may have been released to promote photosynthetic activity and thus increase cell growth. Chae et al. (2020) [47] reported a biostimulatory effect on four different algal species after exposure to expanded polystyrene (EPS, 2 g/L, immersed for 28 days). Responses of *D. magna* after 48 h also increased with time until the effect threshold was exceeded. A higher sensitivity at 48 h compared to 24 h was previously reported by Lithner et al. (2012) [48], who also reported an EC50 value for high-density polyethylene (HDPE) elutriate between 17 and 24 g/L. The study we conducted shows that a much lower concentration (100 mg/L) is able (for PE) to cause a mortality of 20% (glitter H1 and CA7/5) and 40% for another polymer (PMMA), i.e., CA6/1 and C5/1 glitter. Finally, the assay for inhibition of bioluminescence of the bacterium *A. fischeri* showed a response above the effect threshold only in some cases (C5/1, CA6/3, CA6/4, CA6/5), without statistically significant differences in the time factor, but in all cases attributable to the PMMA polymer. The low sensitivity to plastic elutriates in freshwater found by us is consistent with the report of Schiavo et al. (2018) [38], who showed an inhibitory response < 25% after testing elutriates of PE, PP and PS at the same concentration as ours (100 mg/L).

The studies performed with *A. fischeri* but with elutriates in seawater are rarer. For example, Piccardo et al. (2021) [49] reported very little inhibition after 30 min (<15%). The present study showed a more significant response (up to 79.3% at time 3) for PMMA (glitter CA6/4). This discrepancy could be due to the different chemical composition of the particles tested (PE vs. PMMA) as well as the different soaking times (28 vs. 180 days) and total area exposed (16 vs. 30 cm^2^/L). Similar to *R. subcapitata*, the saltwater counterpart *P. tricornutum* also showed good sensitivity to elutriates and frequently exhibited a biostimulatory response. For example, a similar hormesis phenomenon was observed in the marine microalga *Dunaniella tertiolecta* with PE leachate (100 mg/L) [50].

The use of biological assays is a holistic approach that allows the evaluation of toxicity and overall effects of all components, including potential additive, synergistic, and antagonistic effects [51]. Living organisms integrate the positive and negative effects of chemicals with which they come into contact with the environmental conditions to which they are exposed during the experiment and respond to the biologically active components present [52]. Considering the varying sensitivity of organisms to chemicals [53], as well as the overall toxicity of various chemicals released from plastics, the need for a battery of bioassays covering a wide range of trophic levels to assess the toxicity and ecological risks of plastic leachates is evident [54,55]. Organisms from different trophic levels play a fundamental role in maintaining balance in ecosystems, and their characteristics have become an important index for assessing environmental quality [56]. Our study supports this approach. Indeed, the species we used for toxicity testing responded differently to the samples tested. The most sensitive trophic level was that of photosynthesizing primary producers, the second most sensitive species was *A. fischeri*, followed by the larval stages of small planktonic crabs *D. magna* and the plutei of the sea urchin *P. lividus*. The information we collected will therefore support future studies aimed at determining the best test battery for assessing the ecotoxicological risk of plastic elutriates.

Once data have been collected from a series of bioassays, there is a need to further integrate them when a strong contradiction in responses emerges. The importance of integration is that the data can be more easily interpreted to make a risk assessment. In this sense, the TBI approach has gained acceptance in Italy. The data integration used in this study is based on the quantitative assessment proposed by Hartwell, gradually modified by Baudo et al. [37]. It calculates the “ecotoxicological risk” by weighting the type of endpoint observed, the type of environmental matrix analyzed, and the level of agreement of the *t*-test results. This integration confirmed that greater risk was associated with the marine environment, with the highest risk associated with glitter CA6. CA6 comprises hexagonal particles of PMMA. There is a significant knowledge gap regarding the effects of PMMA microplastics and nanoplastics (NP) on aquatic biota [57]. Venâncio et al. (2019) [57] used a series of standard bioassays with four marine microalgae and one marine rotifer species (*Brachionus plicatilis*) to test the toxicity of 40 nm nanoparticles. PMMA-NP was able to induce mortality in rotifers at concentrations greater than 4.69 mg/L, with an estimated 48 h median lethal concentration of 13.27 mg/L. Results collected by Brandts et al. (2021) [58] show that PMMA-NPs activate the antioxidant defenses of gilthead sea bream (*Sparus aurata*) and induce changes in lipid metabolic pathways and genotoxicity in blood cells (40 nm; 0–10 mg/L; 24 h and 96 h exposure). With increasing particle size (1–230 μm), Thomas et al. (2020) [59] demonstrated mild toxicity of PMMA microplastics in early life stages of *Paracentrotus lividus* (no significant increase in developmental defects or in terms of reduced fertilization rate at concentrations of 0.1–10 mg/L). Our study thus lays the first foundations for assessing the ecotoxicological risk of this particular type of plastic polymer, which, although not one of the most produced plastics in Europe (10.7%; [1]), has been shown to be one of the dominant polymers in glitter. A product survey of 37 commercial products containing various glitters found PMMA to be the second most common polymer after PE [60].

Finally, multivariate analysis shows that plastic leachate toxicity is a complex phenomenon, dependent on species sensitivity, in some cases on soaking time and medium (*A. fischeri* in saltwater had greater responses compared to freshwater), and not clearly related with the polymer type. Focusing on the context that led to the worst ecotoxicological outcome (saltwater times 1 and 3), the possible role of contact surface and dye release was explored. In particular, the possible release of dyes was indirectly described by color differences, expressed as Δ*E* and its components (Δ*a*, Δ*b*, Δ*L*). The colorimeter gives the color in the CIEL lab space, which is denoted by L, a and b. L is the luminance, a is the chromaticity and b is the hue. Together they are used to calculate the Δ*E*. Δ*E* indicates how large the color differences are (0 is not perceptible to the human eye, 100 is a completely different color). In some cases, this approach has proven useful. For example, at time 1, the marginal test of the DistLM model showed that the ∆*E* value was statistically significant and could explain 51.6% of the total variability. Such a higher ∆*E* value was associated with a higher proportion of risk: CA6/1 (%*R* = 10.0) and CA6/2 (%*R* = 21.3). Moreover, the brightness variations in CA7/5 (at time 3) were the parameter with the greatest influence on the total difference (∆*E*). Such a variation could be due to the leaching of the surface reflective layer, as shown by the microscopic observations.

According to a recent classification, additives can be divided into four main classes based on their functional and structural components: functional additives, colorants, fillers, and reinforcing agents [61]. Colorants include pigments and azo dyes, which are commonly used to treat textile products [23]. The limited knowledge on the effects of plastic additives often concerns the large category of functional additives, which include stabilizers, flame retardants, antistatics, plasticizers, lubricants, and biocides. For some of these classes of compounds (e.g., plasticizers and flame retardants), there is international legislation aimed at mitigating their effects [23]. However, little is known about the ingredients in the dyes. In the context of glitter, color and shine are key to its success. In fact, glitter is often used in festive contexts to enhance the shine and color of accessories, clothing and makeup. The optical properties that make glitter unique result from the combination of the color variability given by the dyes and the reflectivity given by the metal core. Andrady and Rajapakse (2019) [62] indicate a percentage between 1 and 4% by weight of the polymer for the category of colorants. However, the possible substances included in this category are not known. Consequently, it is also difficult to perform a chemical analysis of the elutriates that could complete the study we conducted. Future studies could focus on chemical characterization techniques of non-target species (since the exact composition of the dyes is not known). In this respect, a chemical screening of non-target species using liquid or gas chromatography coupled with mass spectroscopy could be a good start [63,64].

## 5. Conclusions

The chemical risk posed by a specific category of microparticles, namely glitter, was investigated using two ecotoxicological test batteries (one each for the aquatic, freshwater, and marine environments). Different biological responses (stronger in seawater) and species-specific responses were found, which in some cases also depended on the soaking time. The need to use test batteries for ecotoxicological risk assessment (ERA) of plastic elutriates is thus confirmed, as also pointed out by other authors, including Gao et al. (2022) [55]. The species most sensitive to this form of pollution were algae, followed by the bioluminescent bacterium *A. fischeri*, and then by the primary consumers *D. magna* and *P. lividus*. There is a significant lack of knowledge about the effects of micro- and nanoplastics on aquatic biota, particularly polymers such as poly-methyl-methacrylate (PMMA). Our study therefore lays the initial groundwork for assessing the ecotoxicological risk of this particular type of plastic polymer.

## Figures and Tables

**Table 1:** Characteristics of the nine glitters used to prepare the leachates: sample code, precursor, stereomicroscopic images, shape, mean maximum length and area (expressed in µm and µm^2^ ± standard deviation, respectively), number of particles tested, mean total area for each particle (face A + B), total surface area exposed to water for each type (number of particles * area face A + B, in µm^2^ ± SD) and chemical composition of the outer layer. PMMA = polymethyl methacrylate; PE = polyethylene; PA = polyamide.

Sample Code	Precursor	Photo	Color	Shape	Mean Max Length (µm ± SD)	Mean Area for Face (µm^2^ ± SD)	n.Particles/L Tested	Mean Area Face A+B (µm^2^ ± SD)	Total Surface Exposed to Water (cm^2^/L ± SD)	Chemical Composition of the Outer Layer
CA6/1	purpurine for decorative arts	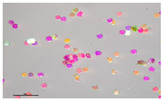	green	hexagonal	221.64 ± 13.48	3.94 × 10^4^ ± 3.44 × 10^3^	3.81 × 10^4^	78,800	30.00	PMMA
CA6/2			purple	hexagonal			3.81 × 10^4^	78,800	30.00	PMMA
CA6/3			orange	hexagonal			3.81 × 10^4^	78,800	30.00	PMMA
CA6/4			pink	hexagonal			3.81 × 10^4^	78,800	30.00	PMMA
CA6/5			yellow	hexagonal			3.81 × 10^4^	78,800	30.00	PMMA
H1	glitter for paint	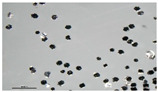	silver	hexagonal	244.66 ± 34.39	4.12 × 10^4^ ± 7.84 × 10^3^	4.48 × 10^4^	82,400	36.87	PE
C5/1	glitter for hair gel	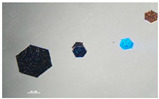	silver	hexagonal	954.09 ± 315.24	6.26 × 10^5^± 3.29 × 10^5^	5.65 × 10^2^	1,252,000	7.08	PMMA
C6/2	glitter for hair gel	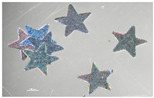	silver	star	3047.83 ± 63.08	3.91 × 10^6^ ± 1.44 × 10^5^	3.41 × 10^2^	7,820,000	26.70	PA
CA7/5	purpurine for decorative arts	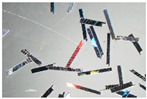	silver	rectangle	2504.22 ± 616.51	8.13 × 10^5^ ± 2.23 × 10^5^	1.92 × 10^3^	1,626,000	31.22	PE

**Table 2:** Results after integration of values from bioassays performed in freshwater: percentage of ecotoxicological risk (%) and the corresponding ecotoxicological risk level (from absent to very high).

	3 Days		90 Days		180 Days	
	%*R*	Ecotoxicological Risk	%*R*	Ecotoxicological Risk	%*R*	Ecotoxicological Risk
C6/2	4.1	absent	4.9	absent	−2.8	absent
C5/1	4.2	absent	2.1	absent	3.1	absent
CA7/5	4.7	absent	3.6	absent	−1.9	absent
H1	3.6	absent	−1.9	absent	−1.7	absent
CA6/1	2.9	absent	0.2	absent	−4.2	absent
CA6/2	−0.4	absent	−0.6	absent	0.0	absent
CA6/3	−2.6	absent	1.8	absent	0.0	absent
CA6/4	6.0	moderate	3.9	absent	−2.6	absent
CA6/5	0.0	absent	8.5	moderate	−1.5	absent
						
		absent	moderate	high	very high	
		≤5%	5–20%	20–50%	>50%	

**Table 3:** Results after integration of values from bioassays performed in saltwater: percentage of ecotoxicological risk (%) and the corresponding ecotoxicological risk level (from absent to very high).

	3 Days		90 Days		180 Days	
	%*R*	Ecotoxicological Risk	%*R*	Ecotoxicological Risk	%*R*	Ecotoxicological Risk
C6/2	−0.7	absent	0.0	absent	0.0	absent
C5/1	−11.5	absent	7.7	moderate	0.0	absent
CA7/5	5.0	moderate	−1.7	absent	15.8	moderate
H1	−1.0	absent	0.0	absent	−8.6	absent
CA6/1	10.0	moderate	−6.1	absent	0.0	absent
CA6/2	21.3	high	1.6	absent	0.0	absent
CA6/3	5.1	moderate	3.3	absent	7.3	moderate
CA6/4	2.0	absent	0.0	absent	18.4	moderate
CA6/5	3.6	absent	5.6	moderate	−10.6	absent
						
		absent	moderate	high	Very high	
		≤5%	5%–20%	20%–50%	>50%	

**Table 4:** Exceeding thresholds for effect, broken down by species and treatment type, with no distinction between different time points. The score can range from a minimum of 0 (white) to a maximum of 3 (red). Green corresponds to a score of 1 and yellow to 2.

	*A. fischeri*	*R. subcapitata*	*D. magna*	*A. fischeri*	*P. tricornutum*	*P. lividus*
C6/2					+	
C5/1		+		+	+	
CA7/5		+			+	
H1		+		+	+	
CA6/1		+			+	
CA6/2					+	
CA6/3		+			+	
CA6/4		+			+	
CA6/5		+			+	

**Table 5:** CIEL lab color differences (D65/2°) between glitter before and after the soaking in saltwater.

	TIME 1				TIME 3			
Samples	∆*E*	∆*L*	∆*a*	∆*b*	∆*E*	∆*L*	∆*a*	∆*b*
CA6/1	36.3	−8.3	7.2	−34.6	16.9	−8.8	−4.1	−13.9
CA6/2	42.4	7.2	−41.7	1.7	30.9	−5.9	−29.7	−6.3
CA6/3	7.5	−1.7	3.8	6.2	15.9	−5.1	8.9	12.2
CA6/4	16.6	1.4	13.3	9.9	14.5	5.7	−1.4	13.2
CA6/5	22.0	3.2	4.5	21.3	12.8	−1.5	11.5	5.5
C6/2	3.3	3.0	−0.8	−1.2	2.9	2.1	1.3	1.5
C5/1	8.9	5.6	−4.7	−5.1	5.7	5.1	1.9	1.5
CA7/5	10.6	10.6	−0.5	−1.0	15.8	15.7	−0.8	0.7
H1	2.6	2.5	−0.4	−0.6	17.0	−7.6	0.5	−15.2

## Data Availability

The data presented in this study are available on request from the corresponding author.

## Supplementary Materials

The following supporting information can be downloaded at: www.mdpi.com/article/10.3390/toxics10110677/s1, Figure S1: CA7/5 type glitter: one of this type of glitter has the typical outer reflective layer, another is completely uncolored; Figure S2: Comparison between the ecotoxicological responses recorded in the two different negative controls: water in plastic bottles only (CN/PL) and in glass bottles (CN/GL); Table S1: Toxicity tests performed on glitter leachates; Table S2: *p* values of 2-way ANOVA, performed to highlight the possible role of the factors TIME and POLYMER in determining the differences in biological responses of saline and freshwater species. Values in bold are statistically significant (<0.05).
