# Supplementary material for: Ecotoxicological Assessment of “Glitter” Leachates in Aquatic Ecosystems: An Integrated Approach
Source: Toxics. 2022 Nov 9;10(11):677. doi: 10.3390/toxics10110677 (PMC9697108; doi:10.3390/toxics10110677)
Supplement: Supplementary file 1 [file toxics-10-00677-s001.zip › toxics-2009328-supplementary.pdf]

Supplementary Materials: Ecotoxicological Assessment of “Glitter” Leachates in Aquatic Ecosystems: An Integrated Approach

M. Piccardo; F. Provenza; S. Anselmi; M. Renzi

ARTIFICIAL SEAWATER (ASW) recipe. Amount given in g/L:

1- $NaCl$ = 22.0
2- $MgCl_2 * 6H_2O$ = 9.7
3- Na_2SO_4 = 3.7
4- $CaCl_2 * 2H_2O$ = 1.32
5- KCl = 0.65
6- $NaHCO_3$ = 0.2
7- H_3BO_3 = 0.023

ARTIFICIAL FRESHWATER (AFW) recipe. Preparation of 4 stock solutions (amount given in g/L):

1- $CaCl_2$ = 11.76
2- $MgSO_4 * 7H_2O$ = 4.93
3- $NaHCO_3$ = 2.59
4- KCl = 0.23

For the final AFW solution, mix 25 mL of each of the previous solutions and keep the volume to 1 L.

Figure S1. CA7/5 type glitter: one of this glitter has the typical outer reflective layer, another is completely uncolored.

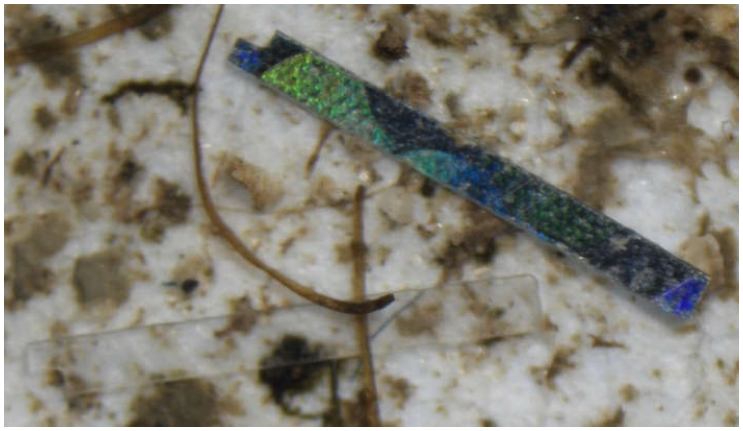

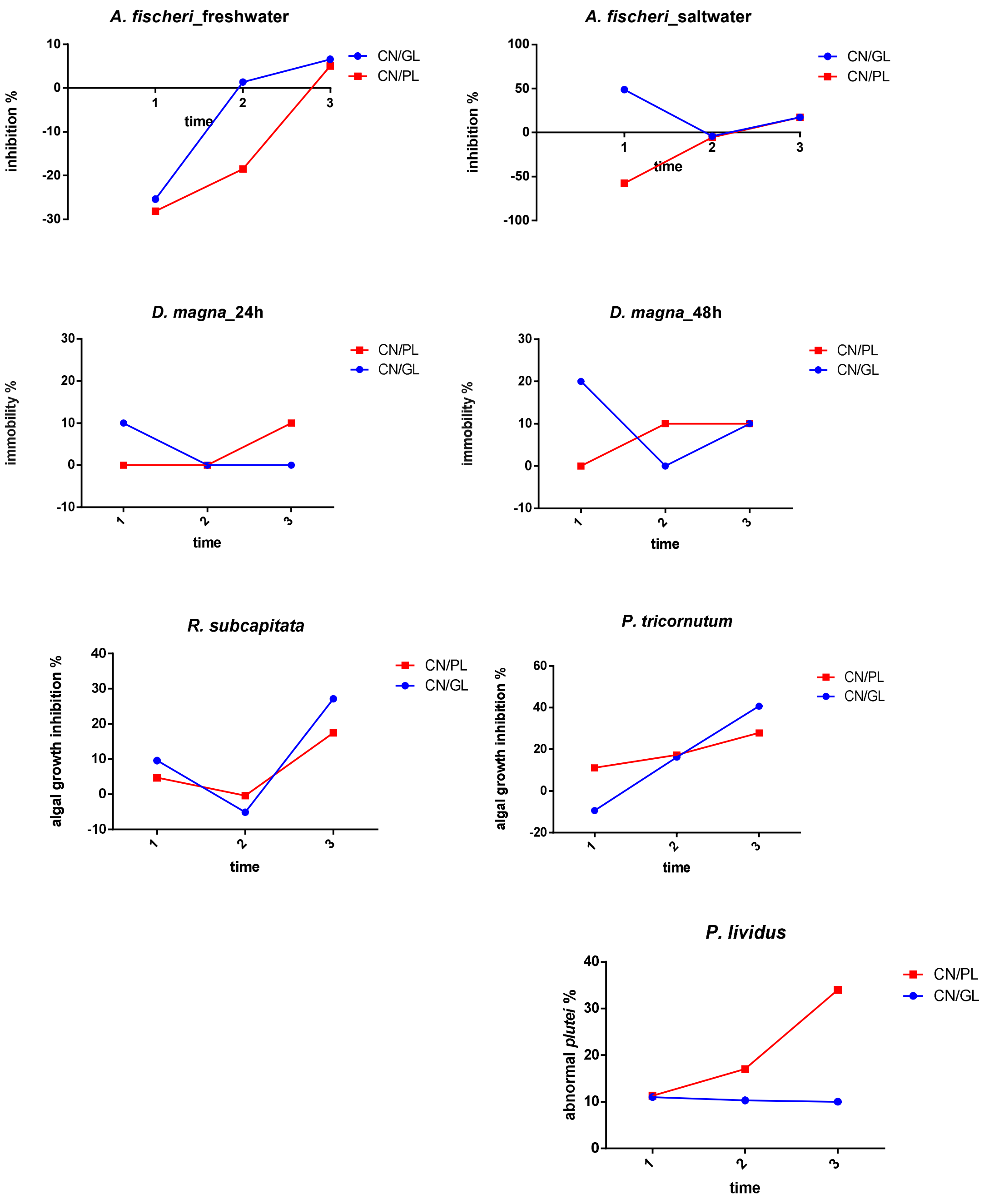

Figure S2. Comparison between the ecotoxicological responses recorded in the two different negative controls: water in plastic bottles only (CN/PL) and in glass bottles (CN/GL).

Table S1. Toxicity tests performed on glitter leachates.

Species		Endpoint	Type	Method	Test duration	Temperature (°C)	Illumination	Aquatic system
Aliivibrio fischeri	Bacteria	Inhibition of bioluminescence	Acute	UNI EN ISO 11348-3:2019	15 minutes 30 minutes	15 ± 1	-	Fresh saltwater
Paracentrotus lividus	Sea urchin	Larval development	Chronic	Chapman et al. 1995 ISPRA Quaderni Ricerca Marina 11/2017	72 hours	18 ± 1	dark	Saltwater
Raphidocelis subcapitata	Algae	Growth inhibition	Chronic	UNI EN ISO 8692:2012	72 hours	20 ± 2	6000-10000 lux	Freshwater
Phaeodactylum tricornutum	Algae	Growth inhibition	Chronic	UNI EN ISO 10253:2017	72 hours	20 ± 2	6000-10000 lux	Saltwater
Daphnia magna	Crustacean	Immobility	Acute	UNI EN ISO 6341:2013	24/48 hours	20 ± 2	dark	Freshwater

Table S2. *P* values of 2-way ANOVA, performed to highlight the possible role of the factors TIME and POLYMER in determining the differences in biological responses of saline and freshwater species. Values in bold are statistically significant (< 0.05).

	**FRESHWATER**			SALTWATER		
	A. fischeri	***R. subcapitata***	***D. magna***	*A. fischeri*	*P. tricornutum*	*P. lividus*
POLYMER	0.8978	0.8507	0.7644	0.0764	0.8063	0.9832
TIME	0.3371	**0.0018**	**0.0296**	0.7078	0.4383	**0.0004**
Interaction	0.8184	0.7591	0.9283	0.3726	0.9831	0.7505
